# First proficiency testing for NGS‐based and combined NGS‐ and FISH‐based detection of *FGFR2* fusions in intrahepatic cholangiocarcinoma

**DOI:** 10.1002/cjp2.308

**Published:** 2023-01-12

**Authors:** Olaf Neumann, Ulrich Lehmann, Stephan Bartels, Nicole Pfarr, Thomas Albrecht, Katharina Ilm, Jens Christmann, Anna‐Lena Volckmar, Hannah Goldschmid, Martina Kirchner, Michael Allgäuer, Maria Walker, Hans Kreipe, Andrea Tannapfel, Wilko Weichert, Peter Schirmacher, Daniel Kazdal, Albrecht Stenzinger

**Affiliations:** ^1^ Institut für Pathologie Universitätsklinikum Heidelberg Heidelberg Germany; ^2^ Institut für Pathologie Medizinische Hochschule Hannover Hannover Germany; ^3^ Institut für Pathologie Technische Universität München Munich Germany; ^4^ German Cancer Consortium (DKTK) Heidelberg Germany; ^5^ Qualitätssicherungs‐Initiative Pathologie, QuIP GmbH Berlin Germany; ^6^ Institut für Pathologie Ruhr Universität Bochum Bochum Germany

**Keywords:** *FGFR2*, fusion, translocation, cholangiocarcinoma, molecular diagnostics, round robin test

## Abstract

Intrahepatic cholangiocarcinoma harbours druggable genetic lesions including *FGFR2* gene fusions. Reliable and accurate detection of these fusions is becoming a critical component of the molecular work‐up, but real‐world data on the performance of fluorescence *in situ* hybridisation (FISH) and targeted RNA‐based next‐generation sequencing (NGS) are very limited. Bridging this gap, we report results of the first round robin test for *FGFR2* fusions in cholangiocarcinoma and contextualise test data with genomic architecture. A cohort of 10 cholangiocarcinoma (4 fusion positive and 6 fusion negative) was tested by the Institute of Pathology, University Hospital Heidelberg, Germany. Data were validated by four academic pathology departments in Germany. Fusion‐positive cases comprised *FGFR2*::*BICC1*, *FGFR2*::*DBP*, *FGFR2*::*TRIM8*, and *FGFR2*::*ATE1* fusions. In a second step, a round robin test involving 21 academic and non‐academic centres testing with RNA‐based NGS approaches was carried out; five participants performed FISH testing in addition. Thirteen of 16 (81%) centres successfully passed the NGS only and 3 of 5 (60%) centres passed the combined NGS + FISH round robin test. Identified obstacles were bioinformatic pipelines not optimised for the detection of *FGFR2* fusions and assays not capable of detecting unknown fusion partners. This study shows the benefit of targeted RNA‐NGS for the detection of *FGFR2* gene fusions. Due to the marked heterogeneity of the genomic architecture of these fusions, fusion partner agnostic (i.e. open) methodological approaches that are capable of identifying yet unknown fusion partners are superior. Furthermore, we highlight pitfalls in subsequent bioinformatic analysis and limitations of FISH‐based tests.

## Introduction

Biliary tract cancer (BTC; cholangiocarcinoma) is the second most common primary liver cancer and accounts for 2% of overall cancer‐related deaths. BTCs are classified according to their anatomical origin into intrahepatic cholangiocarcinoma (iCCA), perihilar cholangiocarcinoma or distal cholangiocarcinoma, and gall bladder carcinoma. While the mutational profiles in these anatomic subtypes partially overlap, iCCA stands out with a high frequency of druggable genetic lesions including *FGFR2* gene fusions. *FGFR2* belongs to a family of receptor tyrosine kinases including the fibroblast growth factor receptors (FGFR1–4). These kinases share a similar structural organisation including an extracellular ligand‐binding domain, which contains three Ig‐like domains, a single‐pass transmembrane segment, a juxta‐membrane domain, a split tyrosine kinase domain, and a short C‐terminal tail. Under normal conditions, the activity of FGFRs is regulated by 18 members of secreted FGFs where ligand engagement leads to dimerisation of the extracellular domain of *FGFR2* and subsequent downstream signalling via RAS‐ERK, PI3K‐AKT, PLC‐g, and STAT [1]. Facilitated through a multitude of different fusion partners, oncogenic ligand‐independent dimerisation of FGFR2 leads to constitutive catalytic activation of FGFR2 [2, 3, 4, 5, 6]. Several phase II studies in *FGFR2‐*fusion‐positive iCCA patients demonstrated consistent efficacy for the pan‐FGFR‐inhibitors pemigatinib, infigratinib, Debio 1347, derazantinib, and futibatinib [7, 8, 9, 10]. Despite the fact that these studies were conducted in pretreated patients, response rates ranged between 21 and 42% and a disease control rate above 80% was achieved. The modified progression‐free survival and modified overall survival ranged from 7 to 9 and 12 to 22 months, respectively. Based on the high efficacy of these drugs in the chemotherapy‐refractory setting, three phase III trials were launched comparing pemigatinib (FIGHT‐302 [11], NCT03656536), infigratinib (PROOF [12], NCT03773302), and futibatinib (FOENIX‐CCA3 [13], NCT04093362) to gemcitabin and cisplatin as the current standard of care as first‐line monotherapy in fusion‐positive iCCA patients. Collectively, these data indicate that testing for *FGFR2* gene fusions is becoming a critical standard of care in patients with cholangiocarcinoma. While the biological underpinnings are clear and clinical exploitability was demonstrated, data on the performance of assays currently used to detect *FGFR2* fusions are far less clear. Extending our comprehensive *in silico* [14] analysis using genomic data from FIGHT‐202 [9], we here report results from a multi‐centric round robin test comparing several RNA‐based next‐generation sequencing (NGS) assays as well as fluorescence *in situ* hybridisation (FISH) and derive testing recommendations.

## Results

### Study design

**Figure 1:**
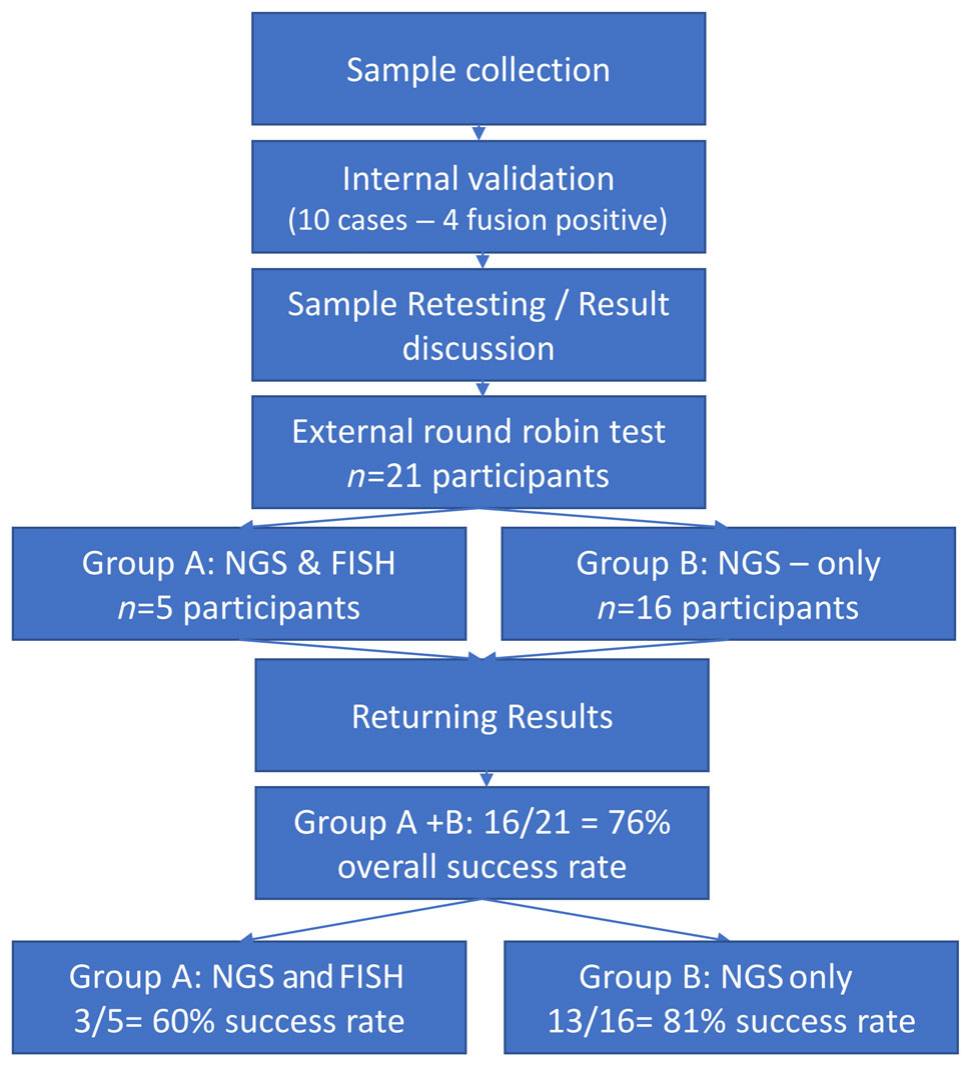
Flow chart of the *FGFR2* round robin test.

The study involved two steps: (1) the internal validation of the results by panel institutes and (2) the external round robin test. Figure 1 depicts the flow chart of the study. The test sets comprised 10 formalin‐fixed and paraffin‐embedded (FFPE) tumour samples of iCCA (tumour cell content >30%). The FFPE material was derived from surgical resection specimens. Four cases were positive for *FGFR2* fusions (Table 1). Fusion‐negative cases harboured other driver mutations known to be mutually exclusive with *FGFR2* fusions [15]. Specifically, all fusion‐negative samples contained *IDH1* activating hot‐spot mutations in Arg132. For each tumour, we supplied one slide for haematoxylin and eosin staining as well as three 5‐μm thick FFPE sections for nucleic acid extraction and two 0.5‐μm thick slides for FISH analysis. The participating centres were blinded to the genetic alterations.

**Table 1:** Tissue samples for NGS and FISH

Case	Alteration	Exons	NGS reference	FISH reference
1	Negative (*IDH1*:p.R132C NM_005896.3)		Negative	Negative
2	*FGFR2*(NM_000141.4)::*BICC1*(NM_011080512.1)	F17B3	Positive	Positive
3	Negative (*IDH1*:p.R132C NM_005896.3)		Negative	Negative
4	*FGFR2*(NM_000141.4)::*TRIM8*(NM_030912.2)	F17T2	Positive	Positive
5	Negative (*IDH1*:p.R132L NM_005896.3)		Negative	Negative
6	Negative (*IDH1*:p.R132C NM_005896.3)		Negative	Negative
7	*FGFR2*(NM_000141.4)::*DBP*(NM_001352.4)	F17D4	Positive	Positive
8	Negative (*IDH1*:p.R132C NM_005896.3)		Negative	Negative
9	Negative (*IDH1*:p.R132L NM_005896.3)		Negative	Negative
10	*FGFR2*(NM_000141.4)::*ATE1*(NM_007041.3)	F17A12	Positive	Negative

The alteration column contains the annotation of the fusion and the exons involved in the rearrangement. Negative cases were chosen with alternative oncogenic drivers that are mutually exclusive with *FGFR2* fusions. The reference values for FISH and NGS depict the correct results for each technique.

The study design of the round robin test allowed for two options: group A: combined FISH and NGS‐testing and group B: NGS‐only testing (see Figure 1). For successful participation in the combined NGS and FISH testing, it was mandatory to pass both tests individually. The maximum achievable score for a test modality (NGS or FISH) was 20 points. For the correct detection of a translocation in the FISH analysis and/or a correct determination of the *FGFR2* fusion status (positive or negative), two points were awarded. Naming of the respective fusion partners was not mandatory. If a detection could not be performed for technical reasons (e.g. limited nucleic acid quality), one point was awarded, though this option could only be used once. The pass score was 90%, which corresponds to 18 points for each part (NGS and FISH). Of note, all targeted NGS assays used RNA as input material. DNA‐based assays were not tested in this study.

### Genomic architecture of *FGFR2* gene fusions

*FGFR2* fusions are characterised by retention of the complete N‐terminal protein (NM_00141: exon 1–17) including an intact kinase domain and by the loss of inhibitory domains (NM_000141: exon 18), which are important for the internalisation of the receptor from the membrane. The gain of dimerisation domains from fusion partners is an additional mechanism that enhances the dimerisation capacities of the fusion proteins and leads to ligand‐independent activation of FGFR2 signalling. Those events are recurrent and well‐described mechanisms of *FGFR2* fusion [16], but most of the fusion transcripts are not yet well characterised due to the high variability of fusion partners and their rare occurrence. For this study, putative fusion transcripts und putative fusion proteins were visualised using the Arriba tool [17].

**Figure 2:**
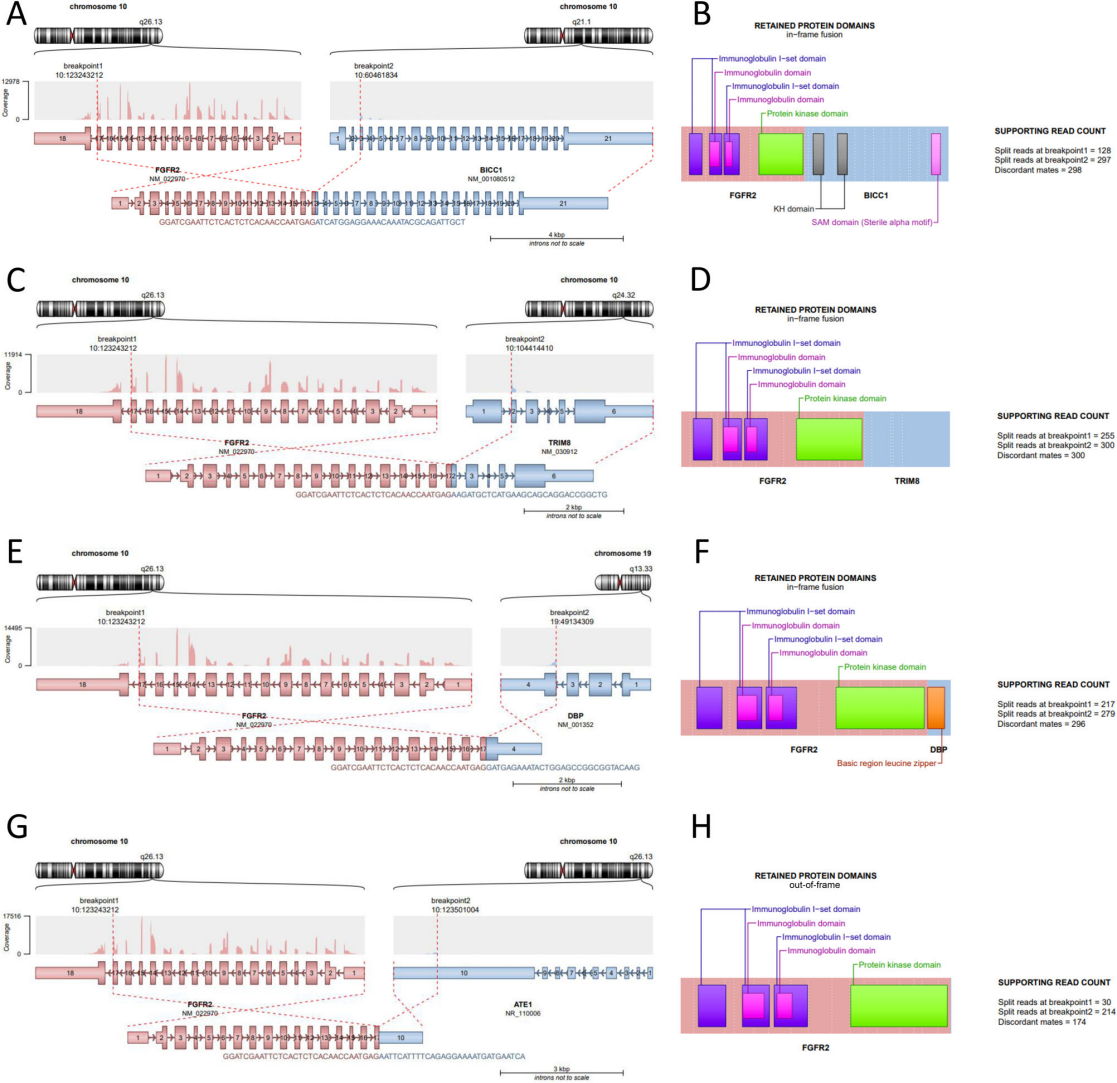
Graphical representation of the *FGFR2* fusion transcripts used and the putative fusion proteins with the encoded domains of both partners joined in the oncoprotein. Left panels: chromosome arms, orientation of the fusion partners, and involved exons in the fusion transcript including read coverage of the exons of the fusion transcript. Right panels: representation of the putative oncofusion protein with the involved domains of both fusion partners and short statistics of the number of reads covering the fusion point. (A) *FGFR2*::*BICC1* fusion transcript. (B) FGFR2::BICC1 oncoprotein. (C) *FGFR2*::*TRIM8* fusion transcript. (D) FGFR2::TRIM8 oncoprotein. (E) *FGFR2*::*DBP* fusion transcript. (F) FGFR2::DBP oncoprotein. (G) *FGFR2*::*ATE1* fusion transcript. (H) FGFR2::ATE1 oncoprotein.

The *FGFR2*::*BICC1* fusion (case 2, Figure 2A,B) is in‐frame and leads to the loss of inhibitory domains in FGFR2 and the gain of a sterile alpha motif domain, which is needed in wild type BICC1 for polymerisation. The fusion of *FGFR2* and *BICC1* (exon 3) is a recurrent and well‐known event.

The *FGFR2*::*TRIM8* fusion (case 4, Figure 2C,D) is in‐frame and leads to the loss of inhibitory domains in FGFR2 and gain of a coiled‐coil domain from TRIM8 which, in wild type TRIM8, is needed for interaction with SOCS1 [18] but can function for dimerisation. *FGFR2*::*TRIM8* fusions were previously identified in the FIGHT‐202 study, but it is unclear whether the same exonic fusion partners were present as the translocation point was only detected in a DNA‐based assay [19].

*FGFR2*::*DBP* fusions (case 7, Figure 2G,H) are described in the scientific literature where *DBP* is a fusion partner with other exons as evident in one case in the FIGHT‐202 trial [9]. This fusion leads to the loss of the inhibitory domains in exon 18 of *FGFR2* and gains a bZIP domain, which is described in the DBP protein as needed for dimerisation with itself or other partners [20].

Case 10 contains a *FGFR2*::*ATE1* fusion (case 10, Figure 2E,F) giving rise to an out‐of‐frame fusion transcript containing only the 3'‐untranslated region (UTR) of the *ATE1* gene. This fusion is defined by the loss of the inhibitory domains/motifs in exon 18 of *FGFR2*. An *FGFR2*::*ATE1* fusion was previously detected in one case of uterine corpus endometrial carcinoma in the TCGA dataset [21] but with other exons fused. As described elsewhere [14], the gene locus of *ATE1* is directly downstream of the *FGFR2* gene locus. This rearrangement leads to a fusion of *FGFR2* and *ATE1* where the fluorochrome‐labelled probes are located further upstream of both *FGFR2* and *ATE1*. This case is challenging because it can hardly be detected using a break‐apart FISH and NGS‐based detection requires specific settings of the bioinformatics pipeline.

### Internal validation

Each case selected and analysed by the lead panel institute (see Table 1) was cross tested by four different academic centres (panel institutes) using a targeted RNA‐based NGS assay. Additionally, two centres employed a FISH assay. Detailed information on extraction methods, sequencing platforms, and assays are provided in supplementary material, Tables S1 and S2. All panel institutes successfully identified and annotated fusion‐positive and fusion‐negative cases.

### External round robin test

Twenty‐one centres participated in the external *FGFR2* round robin test for cholangiocarcinoma. The overview (Figure 1 and supplementary material, Table S3) shows the centres that took part in the group A (NGS and FISH) and group B (NGS‐only test) arms, respectively, as well as the country of origin. All participants reported their results (no dropouts). Supplementary material, Tables S4–S6 list the NGS panels, DNA extraction kits, and bioinformatic pipelines employed by the participating centres, as well as the corresponding success rates.

### NGS and FISH results (group A)

**Figure 3:**
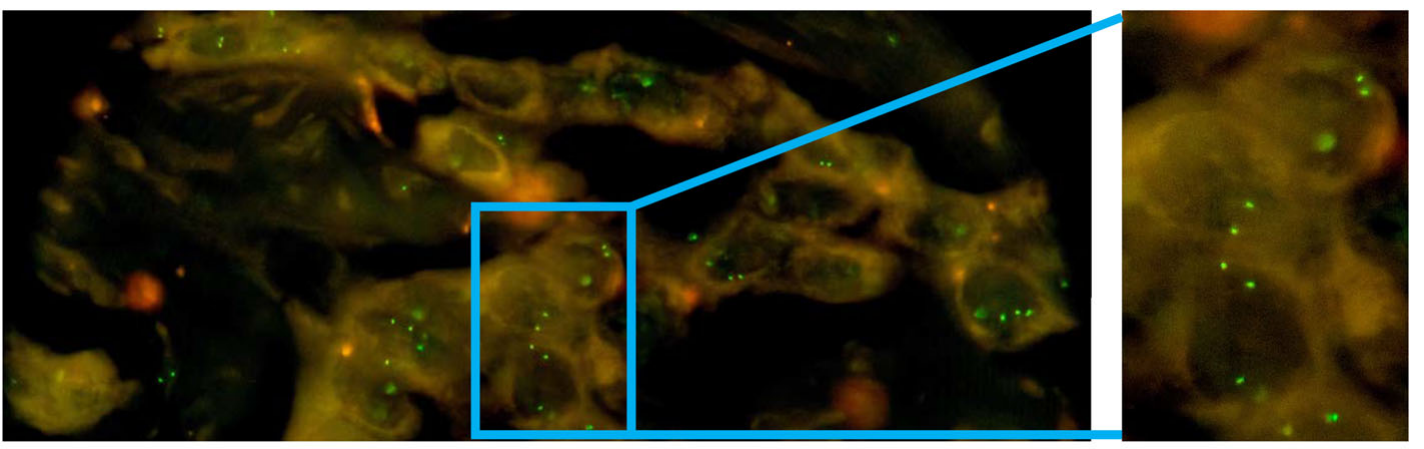
Example image from a panel lab showing the FISH results for the *FGFR2*::*ATE1* fusion. None of the panel labs were able to detect a high‐confidence break‐apart event.

Five centres participated in the combined NGS and FISH arm of the round robin test (group A, see Figure 1). As described above, only successful participation of both the FISH and the NGS parts counted as a pass (see Table 2). All centres submitted their results and were able to correctly detect the fusion‐positive cases by FISH with the exception of case 10 (Table 3). All participants used the ZytoLight SPEC *FGFR2* Dual Color Break Apart Probe (ZytoVision GmbH, Bremerhaven, Germany). The staining of the slides and their analysis were performed manually by all centres in the round robin test. As expected, deviation from the reference was observed for case 10 where the translocation of the partners was difficult to resolve due to the composition of the FISH assay and the genomic architecture of the fusion event (see the description above). None of the four centres involved in the validation part of the study was able to detect a split fusion signal with commercially available FISH probe (exemplified in Figure 3). However, two participating centres rated this case correctly as fusion positive, which might be due to the sensitive detection of subtle split events in individual tumour cells.

**Table 2:** Results of the *FGFR2* round robin test, separated by group and overall

Analysis	Participants	Passed (%)
Group A: FISH + NGS	5	3 (60.0)
Group B: NGS only	16	13 (81.2)
Group A + B	21	16 (76.0)

**Table 3:** Case‐related results of all centres participating in the FISH arm of the study

Case	1	2	3	4	5	6	7	8	9	10
*FGFR2* fusion positive	0	**5**	0	**5**	0	0	**2**	0	0	**2**
*FGFR2* fusion negative	**5**	0	**5**	0	**5**	**5**	**2**	**5**	**5**	**3**
Not evaluated	0	0	0	0	0	0	1	0	0	0

Shaded cells with bold green numbers are the results in accordance with the benchmark result (‘correct’). Bold red numbers show the deviations from the benchmark result.

Case 7 also showed divergent results. Some centres stated that the material quality was suboptimal but, since these FFPE samples are from clinical resection specimens, this reflects a ‘standard scenario’. Since test sets from the slice stacks before and after those used for analysis by the participants performed well in the validation step of this study, there is no reason for excluding this sample based on putative inferior sample quality.

The success rate of the centres in the NGS part of the NGS and FISH arm was 60% (3/5). Forty percentage (2/5) of the institutes, respectively, failed to correctly identify fusions in cases 4 and 7 when using amplicon‐based panels because no primers for the partner gene were included. Sixty percentage (3/5) of the participating centres were not able to correctly analyse case 10. Two out of the five centres participating in the combined NGS and FISH arm failed the NGS part due to using assays (Thermo Fisher OFA assay, AmoyDx HANDLE Classic Panel (Amoy Diagnostics Co. Ltd., Xiamen, China)) that did not contain the primers to detect the *FGFR2*::*DBP* and *FGFR2*::*ATE1* fusions.

### NGS‐only results (group B)

The success rate of the centres in the NGS‐only arm was 81% (13/16) (see Table 2). Table 4 shows the submitted results of the NGS part from all participants; 62.5% (10/16) of the participating centres were not able to correctly analyse case 10. A comprehensive re‐analysis showed that the material is not causative for the deviation from the reference (see Table 1) but rather the NGS panels and technologies used. Case 10 was not removed from the trial but used to illustrate the challenges in *FGFR2* fusion and translocation detection; 6.25% (1/16) and 12.5% (2/16) of the institutes, respectively, failed to correctly identify fusions in cases 4 and 7 when using amplicon‐based panels because no primers for the partner gene were included. Two institutes switched to the Oncomine Comprehensive RNA Assay (OCA) pre‐emptively after not detecting fusions with the Oncomine Focus Assay (OFA). These two centres were then able to detect the *FGFR2*::*BICC1* and the *FGFR2*::*TRIM8* fusion. The participating centre using the OCA plus assay was able to detect the case harbouring a *FGFR2*::*DBP* fusion as ‘fusion positive’ due to an *FGFR2* imbalance test, which is part of the OCA plus assay. Of note, the results of this imbalance assay provide definitive neither evidence regarding the fusion/translocation event nor information on the fusion partner or a preserved reading frame. Therefore, in a real‐world diagnostic scenario, an orthogonal test would be needed to confirm the actual presence of a translocation involving *FGFR2*. The centre using the OCAv3 assay did not detect the *FGFR2*::*DBP* fusion because the assay has neither an imbalance assay for *FGFR2* nor the primers needed to detect the fusion partner *DBP*. Further, the OCAv3 and OCA plus assays were not able to detect the *FGFR2*::*ATE1* fusion due to missing primers for the fusion partner *ATE1*. As described above, the OCAv3 assay lacks an *FGFR2* imbalance assay and the imbalance test of the OCA plus assay failed to identify the gene fusion event.

**Table 4:** Case‐related results of all centres participating in the NGS part of the study

Case	1	2	3	4	5	6	7	8	9	10
*FGFR2* fusion positive	0	**19**	0	**18**	0	0	**17**	**1**	0	**8**
*FGFR2* fusion negative	**21**	**2**	**21**	**2**	**21**	**20**	**4**	**20**	**21**	**13**
Not evaluated	0	0	0	1	0	1	0	0	0	0

Shaded cells with bold green numbers are the results in accordance with the benchmark result (‘correct’). Bold red numbers show the deviations from the benchmark result.

### Identification of the fusion partners and involved exons (groups A and B)

The centres were asked to annotate the detected gene fusions. Precise specification of the fusion event (e.g. fusion partner, involved exons) was not mandatory and therefore not relevant to passing the round robin test. However, as discussed below, this information can be crucial for the functional description and evaluation of the *FGFR2* fusions. Table 5 shows the detected fusions. All participants who were able to detect the fusions also annotated them correctly. The false wild type report for case 7 reflects the issue of a ‘closed’ assay that only detects fusions for which primers are included.

**Table 5:** Overview of the fusions detected in the round robin test

	Fusion partner	Correctly annotated	Incorrectly annotated	False wild type	Not named
Case 2	*FGFR2*::*BICC1*	18	0	2	1
Case 4	*FGFR2*::*TRIM8*	17	0	1	3
Case 7	*FGFR2*::*DBP*	15	0	4	2
Case 10	*FGFR2*::*ATE1*	8	0	13	0

### Summary of test results

Analysis of the results returned by group A (NGS and FISH) revealed that three out of five participants (60%) passed the round robin test. In group B (NGS only), 13 out of 16 participants (81%) passed successfully. The overall success rate of the round robin test was therefore 16 out of 21 participants (76%) (Figure 1 and Table 2).

## Discussion

In this study, we report the results of a multi‐centre round robin test of *FGFR2* fusion testing in cholangiocarcinoma by targeted NGS‐based RNA analysis and FISH. Complementing our *in silico* data [14], the wet‐lab data derived from this study significantly contribute to implementing and optimising *FGFR2* fusion testing in a diagnostic setting.

The detection of full‐length transcripts by RNA‐sequencing in FFPE material is technically challenging due to the prominent degradation of RNA in FFPE samples. Additionally, the genomic architecture of *FGFR2* gene fusions [14] including the diversity of different fusion partners and involved exons needs to be appropriately reflected in a diagnostic test setting. While all methods may be used in routine diagnostics, their ability to detect complex genetic events differs and knowledge of these differences is required for interpretation and reporting of test results. Broadly, one can differentiate between ‘closed’ assays, e.g. amplicon‐based assays, which rely on specific primers targeting *FGFR2* and the corresponding specific exons of the partner genes, and partner‐agnostic assays (e.g. hybrid capture, single primer extension), which are – by design – able to detect known and unknown (novel) fusion partners [14, 22].

The OFA is not suitable to detect any of the gene fusions used in this round robin test. While primers for *BICC1*, one of the fusion partners of the *FGFR2* fusion‐positive cases used in this round robin test, are indeed part of the assay design, they are currently restricted to *BICC1* exon 2. In contrast, due to the more comprehensive design of the test, the OCAv3/plus RNA assay is able to detect the *FGFR2*::*BICC1* and *FGFR2*::*TRIM8* fusions correctly. While the OCA plus assay also contains primers targeting *ATE1*, it is unable to detect the *FGFR2*::*ATE1* fusion present in case 10 due to missing primers for the respective exon involved in the fusion. *DBP* as a fusion partner is not covered by the OCAv3/plus assay at all. Similarly, due to its design, the AmoyDx HANDLE Classic assay can detect the *FGFR2*::*BICC1* fusion present in one of the cases, but it is unable (due to missing primers) to identify *DBP*, *TRIM8*, or *ATE1* as fusion partners. These observations are well in line with our *in silico* analysis published recently [14] and indicate that practical considerations of the assay design and interpretation of the results should primarily be based on the precise knowledge of the genomic architecture of *FGFR2* fusions. Based on these foundations, diagnostic laboratories may balance technical pros and cons of each of those tests, which collectively drive decisions regarding interpretation and selection of the right approach for detection of *FGFR2* fusions.

One of the participating centres employing a fusion partner agnostic (i.e. open) NGS‐based assay (QIAseq Multimodal Custom Panel (Qiagen GmbH, Hilden, Germany)) reported a deviating result for case 10. While the design of the assay enables the detection of *FGFR2*::*ATE1*, its calling is influenced by the bioinformatics pipeline. Specifically, the fusion point of *ATE1* was localised in the 3′‐UTR. Therefore, no coding exons were involved in the fusion event nor was an in‐frame fusion generated. By default, which intentionally keeps the number of false positive fusions low, this setting led to the filtering out of this event by the bioinformatics pipeline. Since *FGFR2* fusions can be, but do not necessarily have to be, in‐frame to unfold their oncogenic potential, it is necessary to be able to detect intra‐exonic fusions. The default settings of the Archer and Qiagen pipelines can be modified by permitting the call of exonic fusion events. After recalibration of the bioinformatics pipeline, the fusion in case 10 was correctly identified. This special case exemplifies the importance of being aware of the bioinformatic steps in the diagnostic workflow.

FISH break‐apart analysis can determine the break in the *FGFR2* gene but provides no information on the fusion partner nor information regarding the potential functionality of the fusion (i.e. ‘in‐frame’ status). As shown in this study, depending on the specific genomic architecture, FISH analyses may even miss fusion events.

In summary, in‐depth knowledge of the genomic architecture as well as the assay design is crucial to reliably detect *FGFR2* fusions in iCCA. While all assays may be used, their advantages and limitations should be considered for implementation, testing, and reporting.

## Author contributions statement

ON, TA, ALV, HG, MK, MA, PS, DK and AS conceived and carried out collection of samples, experiments, and analysed data. UL, SB, NP, JC, MW, HK, AT and WW carried out experiments and analysed data. KI analysed data. All authors were involved in writing the paper and had final approval of the submitted and published versions.

## Supporting information


**Table S1.** Method selection of lead panel and panel institutes for *FGFR2* fusion testing (next‐generation sequencing [NGS])
**Table S2.** Fluorescence *in situ* hybridisation (FISH) probes and analysis methods for the internal proficiency *FGFR2* FISH testing
**Table S3.** Overview of the test sets and country of origin of participants
**Table S4.** NGS panels used by the institutes in the external round robin test
**Table S5.** RNA extraction kits used by the centres in the external round robin test
**Table S6.** Analysis pipelines/software used by the centres in the external round robin test

## Data Availability

The genetic data can be made available upon request.
