# Supplementary material for: First proficiency testing for NGS‐based and combined NGS‐ and FISH‐based detection of FGFR2 fusions in intrahepatic cholangiocarcinoma
Source: J Pathol Clin Res. 2023 Jan 12;9(2):100–7. doi: 10.1002/cjp2.308 (PMC9896158; doi:10.1002/cjp2.308)
Supplement: Supplementary file 1 — Table S1. Method selection of lead panel and panel institutes for FGFR2 fusion testing (next‐generation sequencing [NGS]) Table S2. Fluorescence in situ hybridisation (FISH) probes and analysis methods for the internal proficiency FGFR2 FISH testing Table S3. Overview of the test sets and country of origin of participants Table S4. NGS panels used by the institutes in the external round robin test Table S5. RNA extraction kits used by the centres in the external round robin test Table S6. Analysis pipelines/software used by the centres in the external round robin test [file CJP2-9-100-s001.pdf]

First proficiency testing for NGS-based and combined NGS- and FISH-based detection of *FGFR2* fusions in intrahepatic cholangiocarcinoma

O Neumann *et al. J Pathol Clin Res,* https://doi.org/10.1002/cjp2.308

Supplementary Tables

Table S1. Method selection of lead panel and panel institutes for *FGFR2* fusion testing (next-generation sequencing [NGS])

Table S2. Fluorescence *in situ* hybridisation (FISH) probes and analysis methods for the internal proficiency *FGFR2* FISH testing

Table S3. Overview of the test sets and country of origin of participants

Table S4. NGS panels used by the institutes in the external round robin test

Table S5. RNA extraction kits used by the centres in the external round robin test

Table S6. Analysis pipelines/software used by the centres in the external round robin test

Table S1. Method selection of lead panel and panel institutes for *FGFR2* fusion testing (NGS)

Lab	Nucleic acid extraction	Concentration determination	Detection method	Platform	NGS Panel	Analysis software
**Lead**	Maxwell® RSC RNA FFPE Kit (Promega)	Qubit (Thermo Fisher)	NGS	Ion GeneStudio S5/S5P (Thermo Fisher)	Archer® FusionPlex Lung panel (ArcherDx)	Archer Analysis 6.2.3
**1**	Maxwell® RSC RNA FFPE Kit (Promega)	Qubit (Thermo Fisher)	NGS	Ion GeneStudio S5 (Thermo Fisher)	Oncomine Comprehensive Assay v3 (Thermo Fisher) +Archer® FusionPlex Lung panel (ArcherDx)	Ion Reporter (Thermo Fisher)
**2**	Maxwell® 16 LEV RNA FFPE Purification Kit (Promega)	Not performed	NGS	NextSeq (Illumina)	QIAseq Multimodal Custom panel (Qiagen)	CLC Genomics Workbench (Qiagen)
**3**	Maxwell® 16 LEV RNA FFPE Purification Kit (Promega)	Qubit (Thermo Fisher)	NGS	MiSeq (Illumina)	QIAseq Targeted RNA Custom Panels (Qiagen)	Seamless NGS (ecSeq Bioinforma-tics GmbH)
**4**	Maxwell® RSC RNA FFPE Kit (Promega)	Qubit (Thermo Fisher)	NGS	NextSeq (Illumina)	TruSight Oncology 500 (Illumina)	TSO500 RUO 2.2.0.12 (Illumina)

Table S2. FISH probes and analysis methods for the internal proficiency *FGFR2* FISH testing

Lab	Method	Platform	Probes	Analysis method
**Lead**	FISH	Manual staining	ZytoLight ® SPEC *FGFR2*/CEN 10 Dual Color Probe (ZytoVision GmbH)	Manual analysis
**1**	FISH	Manual staining	ZytoLight ® SPEC *FGFR2* Dual Color Break Apart Probe (ZytoVision GmbH)	Manual analysis
**2**	FISH	Manual staining	ZytoLight ® SPEC *FGFR2* Dual Color Break Apart Probe (ZytoVision GmbH)	Computer-based analysis system – Allegro Plus System (Bioview)

Table S3. Overview of the test sets and country of origin of participants

**Location of participating institutions**	**Quantity of test sets requested for**	
	**NGS**	**NGS and FISH**
Germany	15	4
Austria	1	1
**In total**	**16**	**5**

Table S4. NGS panels used by the institutes in the external round robin test

Manufacturer	Kit/Panel	Number of Participants	Success rate (%)
AmoyDx	HANDLE Classic NGS Panel	1	0 (0)
	HANDLE Classic NGS Panel + AmoyDx Comprehensive Panel	2	2 (100)
ArcherDx	FusionPlex Lung	5	5 (100)
	FusionPlex Expanded Lung	2	2 (100)
Illumina	TruSight Oncology 500	1	1 (100)
	TruSight Tumor 15	1	1 (100)
	TruSight RNA-Fusion	2	2 (100)
	TruSight Tumor 170	1	1 (100)
Qiagen	QIAseq Multimodal Custom Panels	1	1 (100)
Sophia Genetics	Custom RNA target Technology Solution	1	1 (100)
Thermo Fisher	Oncomine Focus Assay	2	0 (0)
	Oncomine Focus Assay + Oncomine Comprehensive Assay Plus	2	0 (0)

Table S5. RNA extraction kits used by the centres in the external round robin test

Manufacturer	Extraction Kit	Number of Participants	Success rate (%)
Promega	Maxwell® RSC RNA FFPE Kit	8	8 (100)
	Maxwell® 16 LEV RNA FFPE Purification Kit	2	2 (100)
Qiagen	RNeasy FFPE Kit	4	3 (75)
	AllPrep DNA/RNA FFPE Kit	1	1 (100)
Thermo Fisher	RecoverAll Total Nucleic Acid Isolation Kit	3	1 (33)
AmoyDx	FFPE DNA/RNA Kit	1	1 (100)
Roche	MagNA Pure Compact RNA Isolation Kit	1	0 (0)
ZymoResearch	Quick-RNA 96 Kit	1	0 (0)

Table S6. Analysis pipelines/software used by the centres in the external round robin test

**Manufacturer**	**Software**	**Number of Participants**	**Success rate (%)**
ArcherDx	Archer Analysis	7	7 (100)
AmoyDx	ANDAS Data Analyzer	3	2 (67)
Illumina	TruSight Tumor 170 BaseSpace app	1	1 (100)
	Local Run Manager	1	1 (100)
PierianDx	PierianDx	1	1 (100)
Qiagen	CLC Genomics Workbench	3	3 (100)
Sophia Genetics	SOPHiA DDM™	1	1 (100)
Thermo Fisher	Ion Reporter	3	0 (0)
Thermo Fisher + Sophia Genetics	Ion Reporter + SOPHiA DDM™	1	0 (0)
